# Emerging ecophenotype: reward anticipation is linked to high-risk behaviours after sexual abuse

**DOI:** 10.1093/scan/nsac030

**Published:** 2022-04-19

**Authors:** Pia Pechtel, Jennifer Harris, Anke Karl, Caroline Clunies-Ross, Susie Bower, Nicholas J Moberly, Diego A Pizzagalli, Edward R Watkins

**Affiliations:** Department of Psychology, College of Life and Environmental Sciences (CLES), University of Exeter, Exeter EX4 4QG, UK; Department of Psychology, College of Life and Environmental Sciences (CLES), University of Exeter, Exeter EX4 4QG, UK; Department of Psychology, College of Life and Environmental Sciences (CLES), University of Exeter, Exeter EX4 4QG, UK; Child and Adolescent Mental Health Services, Children and Family Health Devon, Exeter EX2 4NU, UK; Child and Adolescent Mental Health Services, Children and Family Health Devon, Exeter EX2 4NU, UK; Department of Psychology, College of Life and Environmental Sciences (CLES), University of Exeter, Exeter EX4 4QG, UK; Center for Depression, Anxiety and Stress Research, McLean Hospital, Belmont MA 02478, USA; Department of Psychiatry, Harvard Medical School, Boston, MA 02215, USA; McLean Imaging Center, McLean Hospital, Belmont, MA 02478 USA; Department of Psychology, College of Life and Environmental Sciences (CLES), University of Exeter, Exeter EX4 4QG, UK

**Keywords:** sexual abuse, high-risk behaviour, reward, adolescence, fMRI

## Abstract

Adolescents frequently engage in high-risk behaviours (HRB) following childhood sexual abuse (CSA). Aberrant reward processes are implicated in HRB, and their underlying fronto-striatal networks are vulnerable to neurodevelopmental changes during adversity representing a promising candidate for understanding links between CSA and HRB. We examined whether fronto-striatal responses during reward anticipation and feedback (i) are altered in depressed adolescents with CSA compared to depressed, non-abused peers and (ii) moderate the relationship between CSA and HRB irrespective of depression. Forty-eight female adolescents {14 with CSA and depression [CSA +  major depressive disorder (MDD)]; 17 with MDD but no CSA (MDD); 17 healthy, non-abused controls} completed a monetary reward task during functional magnetic resonance imaging. No differences in fronto-striatal response to reward emerged between CSA + MDD and MDD. Critically, high left nucleus accumbens activation during reward anticipation was associated with greater HRB in CSA + MDD compared to MDD and controls. *Low* left putamen activation during reward feedback was associated with the absence of HRB in CSA + MDD compared to MDD. Striatal reward responses appear to play a key role in HRB for adolescents with CSA irrespective of depression, providing initial support for a *CSA ecophenotype*. Such information is pivotal to identify at-risk youth and prevent HRB in adolescents after CSA.

**Key Points**

- Sexual abuse can affect the neurodevelopment of fronto-striatal reward networks and is a risk factor for high-risk behaviours.
- We investigated if reward-based neural alterations specifically linked to sexual abuse may form a mechanism underlying high-risk behaviours irrespective of depression.
- Aberrant striatal responses to reward were linked to high-risk behaviours in depressed adolescents with sexual abuse but not their non-abused peers matched for depression or healthy controls. Striatal reward responses appear to be critically implicated in high-risk behaviours after sexual abuse irrespective of depression.
- The same sexual abuse ecophenotypic variations that form a *vulnerability* to high-risk behaviours (HRB) may be utilized as *assets* in clinical interventions to prevent HRB following sexual abuse (e.g. heightened sensitivity to prosocial rewards).

Childhood sexual abuse (CSA) is reported by ∼7.5% of UK adults. Police recorded CSA offences increased by 267% from 2013 to 2020 and CSA-related calls to helplines increased by 3-fold during coronavirus disease 2019 (COVID-19) restrictions (Office for National Statistics, 2020). Rapid rises in prevalence make research on the sequelae of CSA of urgent public health significance.

Adolescence is a time of increased sensation seeking often manifested in greater risk-taking which is considered a part of normative development (Dahl, 2004; Steinberg, 2008). Yet, some young people use extreme or multiple HRB to relieve intense, negative affect (i.e. negative reinforcement) that can cause serious harm or increase their vulnerability to exploitation. For example, exposure to CSA is a significant risk factor for HRB such as non-suicidal self-injury (Serafini *et al.*, 2017), sex as self-injury (Zetterqvist *et al.*, 2018), unsafe sexual behaviours (Thibodeau *et al.*, 2017), substance misuse (Kisely *et al.*, 2021), disordered eating (Wonderlich *et al.*, 1997) or aggressive behaviours (Braga *et al.*, 2017). Despite providing short-term emotional relief, long-term consequences of HRB can include revictimization (Herbert *et al.*, 2020), sexual exploitation (Laird *et al.*, 2020) and disruption in brain development (Hoops *et al.*, 2018).

Although there is variability in the types of HRB that are used by young people after CSA, various HRB commonly co-occur (Warne *et al.*, 2021) and have been suggested to share a similar function of negative reinforcement (e.g. Klonsky, 2007; Dingemans *et al*., 2017). To date, the majority of individual- and family-level interventions for HRB show no or little improvement (MacArthur *et al.*, 2018). Only a few interventions show small effects for specific HRB (e.g. self-harm; Kothgassner *et al.*, 2020). One reason could be that interventions do not explicitly consider the neurobiological changes associated with adversity, also known as ecophenotypic variation (Teicher and Samson, 2016) and for which HRB may be particularly effective as relief. Consequently, identifying mechanisms predicting HRB in those with CSA can help to recognize vulnerable youth and tailor clinical interventions to avoid cycles of negative reinforcement.

The fronto-striatal reward circuit is a promising candidate for understanding links between CSA and HRB. Fronto-striatal regions undergo considerable changes in adolescence, making them susceptible to disrupted development due to excessive glucocorticoid release expected to co-occur with CSA (Braams *et al.*, 2015; Pechtel and Pizzagalli, 2011). It is important to note that changes in fronto-striatal reward function are also related to major depressive disorder (MDD) (Keren *et al.*, 2018; Xie *et al.*, 2021) which in turn is a common sequel of CSA (Teicher *et al*., 2009). Accordingly, to parse out the effect of CSA and depression in fronto-striatal reward functioning, the current study includes young people with CSA and MDD and those with MDD but no CSA.

Striatal and prefrontal cortex (PFC) development is vulnerable to adversity from birth to around age 9 and 14–16 years, respectively (Andersen *et al.*, 2008; Hanson *et al.*, 2016). Although considered adaptive in the context of CSA (i.e. increasing chances of survival), neural changes may be maintained when the person transitions from an adverse to a safe environment, thereby increasing the risk of psychopathology and the need for HRB to manage affect (McCrory and Viding, 2015; Teicher *et al.*, 2016).

The striatum plays a critical role in motivational (‘*wanting*’) and hedonic (‘*liking*’) aspects of reward (DePasque and Galván, 2017). However, variable activation patterns are reported for HRB, adversity, or depression, preventing translation into therapeutic approaches. For example, mixed findings of increased and decreased striatal activation during reward anticipation and reward feedback have been found in non-abused adults engaging in HRB (see Balodis and Potenza, 2020 for a review). Studies in adolescents with specific HRB showed heightened activation in the bilateral putamen in response to a monetary reward (Poon *et al.*, 2019). Following adversity, diminished striatal response to both anticipation and feedback of reward has been demonstrated in children (Mehta *et al.*, 2010; Takiguchi *et al.*, 2015), adolescents (Hanson *et al.*, 2015) and adults (Dillon *et al.*, 2009; Hanson *et al.*, 2016). Adolescents with depression show blunted striatal and orbitofrontal cortex (OFC) activation and increased medial PFC (mPFC) activation to reward anticipation and feedback (Forbes *et al.*, 2009; Keren *et al.*, 2018; Xie *et al.*, 2021). Despite CSA being a risk factor for HRB and depression, it remains unclear how fronto-striatal responses to reward would be expressed in depressed adolescents with CSA who are relying on HRB to cope.

One possible explanation for varying patterns in reward processing is ecophenotypic variations following childhood adversity. The *maltreatment ecophenotype* highlights that abused and non-abused individuals with matching clinical presentations differ in clinical, neurobiological and genetic factors as an adaptation to environmental conditions (Teicher and Samson, 2016), as well as in their response to treatment (Nanni *et al.*, 2012). However, specific types of maltreatment have also been shown to have varying impact on psychopathology and brain development (Teicher *et al.*, 2016). Indeed, unlike other forms of abuse, CSA in particular predicts an earlier onset of depression (Teicher *et al*., 2009), greater use of HRB (Serafini *et al.*, 2017; Tatnell *et al.*, 2017) and blunted reward learning (Pechtel and Pizzagalli, 2013). To date, examining reward-based characteristics of a CSA ecophenotype as a potential mechanism for HRB remains unexplored.

To fill this gap, we investigated whether fronto-striatal responses during reward anticipation and feedback (i) are altered in adolescents with CSA and MDD compared to those with MDD only and non-abused, healthy controls and (ii) moderate the relationship between CSA and HRB irrespective of depression. We expected that depressed adolescents with CSA would show distinct ecophenotypic variations in response to reward compared to those with depression only and controls and that altered fronto-striatal responses would moderate the relationship between CSA and HRB compared to non-abused, depressed peers. To investigate this, we examined fronto-striatal activation in regions of interest (ROIs) implicated in reward processing across key publications on depression, HRB and adversity including nucleus accumbens (NAcc; Braams *et al.*, 2015), caudate (Keren *et al.*, 2018), pallidum (Dillon *et al.*, 2009), putamen (Poon *et al.*, 2019), mPFC (Forbes *et al.*, 2010), OFC (Xie *et al.*, 2021) and ventrolateral PFC (vlPFC; Ambrosia *et al.*, 2018; Table S1; Figure S1).

## Methods

### Participants

Fifty-two adolescents (aged 13–19 years) were recruited from the community and mental health services. Two participants did not complete the study (CSA: *n *= 2), and two participants generated incomplete data sets (MDD: *n* = 1; CSA: *n* = 1). The final data set (*N* = 48; M_age_ = 17.04 years; s.d. = 1.79) consisted of 14 females with CSA and current MDD (CSA + MDD), 17 females with current MDD but no CSA (MDD) and 17 healthy, non-abused females (controls).

The CSA + MDD group reported at least one incident of coerced sexual contact as indicated by the Child Trauma Questionnaire (CTQ: sexual abuse ≥6; Bernstein *et al.*, 1994). The CSA + MDD and MDD groups met diagnostic criteria for current major depression, as assessed by the Kiddie-Schedule for Affective Disorders and Schizophrenia (KSADS; Kaufman *et al.*, 1997) administered by psychologists. Clinical groups were matched for the number of past MDD episodes, exposure to treatment and comorbid anxiety presentations. Controls did not report any current or past psychiatric disorders or history abuse as indicated by the CTQ and the KSADS. The study was approved by the UK Health Research Authority. Participants and guardians provided informed consent according to the Declaration of Helsinki.

### Measures

The Beck Depression Inventory-II (Beck *et al.*, 1996), the Snaith–Hamilton Pleasure Scale (Snaith *et al.*, 1995) and a five-item Brooding subscale from the Ruminative Response Scale (Treynor *et al.*, 2003) assessed aspects of depression. The Risky Behaviour Questionnaire (RBQ; Auerbach and Gardiner, 2012) recorded the frequency of HRB in the past month (e.g. self-harm, substance misuse and aggression). Self-reported measures of Positive and Negative Affect Schedule - Positive (PANAS-P) and Positive and Negative Affect Schedule - Negative (PANAS-N; Watson *et al.*, 1988) were completed immediately before and after the scan.

In the scanner, participants completed the card-guessing task (∼20 min), which has been used to probe reward-related brain activation in young people with depression and HRB (Forbes *et al.*, 2009; Poon *et al.*, 2019). Participants guessed if an unknown card with a possible value of one to nine was higher or lower than five to yield a real monetary reward with predetermined, equal outcomes for all participants (Figure S2). The sequence of events in each trial was as follows: guess (4 s), trial (reward or loss; 8–12 s), number (0.5 s), feedback (reward, loss or no change; 0.5 s) and wait (7–11 s). The total of 48 trials was comprised of 12 win-reward, 12 win-no-change, 12 loss-loss and 12 loss-no-change. Reaction times and the number of missed trials were recorded to determine task compliance (Table S2).

### Data acquisition and analyses

Group comparisons were completed using ANOVA and *post hoc* Fisher’s least significant difference. The Greenhouse–Geisser correction was used where appropriate. Due to group differences, CTQ emotional and physical abuse subscales scores were added as covariates. Functional images were acquired on a Philips 1.5 T scanner using an Echo-planar imaging sequence, with TR/TE = 3,000/45 ms; 388 volumes; field of view = 240 mm; Matrix = 80 × 80; voxel size = 3 × 3 × 3 mm; flip angle = 90° and 38 slices. Neuroimaging analyses used Statistical Parametric Mapping (SPM) software (Version 12, http://www.fil.ion.ucl.ac.uk/spm) and the Artifact Detection Toolbox (Version 2015-10; https://www.nitrc.org/projects/artifact_detect). To increase replicability, general linear models were based on previous publications (Caseras *et al.*, 2013). Main effects were generated for *reward anticipation vs baseline* and *reward feedback of win trials vs baseline.* The MarsBar toolbox for SPM was used to extract beta weights (parameter estimates of activation; arbitrary units) from each subject averaged across all voxels within each ROI for the reward anticipation and reward feedback contrasts as part of the second (group) level functional magnetic resonance imaging (fMRI) analysis. Values were added to Statistical Package for the Social Sciences prior to analysing fronto-striatal group differences (Aim 1) and moderation models (Aim 2).

Multivariate analysis of covariance for striatal (bilateral: NAcc, caudate, pallidum and putamen) and prefrontal (mPFC, OFC and bilateral vlPFC) regions determined group-level main effects (anticipation and feedback) with covariates (physical and emotional abuse). Moderate correlations were reported among dependent variables (Tables S3 and S4). ROI analyses used the MarsBar toolbox (*P* < 0.05, peak family-wise error and cluster false discovery rate corrected; http://marsbar.sourceforge.net/; Brett *et al.*, 2002; see Supporting Information for additional methods).

Simple moderator analyses were performed as hierarchical regressions using PROCESS (Hayes, 2013). The multi-categorical variable of ‘group’ (Helmert coded as *k − *1 groups*: clinical groups vs controls* and *CSA + MDD vs MDD*) served as predictor for HRB (RBQ scores). Mean-centred beta weights were entered as moderators in separate models (one per ROI). To test for moderation, the group interaction model (*clinical groups vs controls × ROI beta weights* and *CSA + MDD vs MDD × ROI beta weights*) was compared to the model without this interaction. A statistically significant increase in *R*^2^ when the group interaction is added constitutes affirmative evidence for moderation. Simple slope analysis using the omnibus interference test probed the interactions and visualized the models (Hayes and Montoya, 2017). Instead of grouping participants based on their actual beta weights values, the statistics represent differences in the slopes by creating slopes as if everyone was considered low, average or high in beta values.

## Results

Participants showed no group differences in age or ethnicity. Clinical groups (CSA + MDD and MDD) were matched for number of episodes, past or current therapeutic treatment and numbers of current anxiety comorbidity (Table 1; Table S5).

**Table 1.** Demographic and clinical data

	CSA + MDD	MDD	Controls	χ^2^/*t*/*F*-		
	(*n *= 14)	(*n *= 17)	(*n *= 17)	value	*P*-value	η_p_^2^
Demographics						
Age, M (s.d.)	17.43 (1.56)	17.35 (1.77)	16.41 (1.91)	1.69	0.20	0.07
Ethnicity: Caucasian, no. (%)	13 (92.9)	16 (94.1)	13 (76.5)	2.94	0.23	
Treatment: past, no. (%)	10 (71.4)	11 (64.7)	–	–	0.50^a^	
Treatment: current, no. (%)	10 (71.4)	10 (58.8)	–	–	0.36^a^	
No. MDD episodes, M (s.d.)	2.43 (0.85)	1.94 (0.83)	–	1.61	0.12	0.58
Single episode, no. (%)	2 (14.3)	6 (35.3)	–		0.18^a^	
Recurrent episodes, no. (%)	12 (85.7)	11 (64.7)			0.18^a^	
Current anxiety Dx, no. (%)	5 (35.7)	7 (41.2)	–	–	0.53^a^	
Clinical measures						
CTQ—emotional, M (s.d.)	12.71 (7.19)^b^	10.82 (5.51)^c^	6.29 (1.76)	6.43	0.003	0.22
CTQ—physical, M (s.d.)	8.23 (4.73)^b^	7.06 (3.90)	5.12 (0.33)	3.62	0.04	0.14
CTQ—sexual, M (s.d.)	14.29 (7.42)^d^	5.00 (0.00)	5.00 (0.00)	26.92	<0.001	0.54
BDI-II, M (s.d.)	27.00 (9.53)^e^	28.47 (9.40)^f^	4.82 (3.52)	46.86	<0.001	0.68
SHAPS, M (s.d.)	27.50 (4.52)^e^	30.35 (6.44)^f^	20.53 (3.28)	17.61	<0.001	0.44
Brooding, M (s.d.)	15.21 (3.27)^e^	15.41 (2.58)^f^	8.35 (2.03)	38.69	<0.001	0.63
RBQ, M (s.d.)	9.64 (6.61)^b^	9.71 (6.01)^c^	4.65 (4.50)	4.26	<0.02	0.16

Notes. M = mean; s.d. = standard deviation, Dx = diagnosis; BDI-II = Beck Depression Inventory-II; No. = Number; SHAPS = Snaith–Hamilton Pleasure Scale; CSA+MDD = adolescents with history of childhood sexual abuse and current depression; MDD = adolescents with current depression but no history of childhood sexual abuse.

a Fisher’s exact test (one-tailed);

b CSA + MDD and controls significantly differ [*P* < 0.05, Fisher’s least significant difference (LSD)].

c MDD and controls significantly differ (*P* < 0.05; LSD).

d CSA + MDD significantly differ from MDD and controls (*P* < 0.001; LSD).

e CSA + MDD and controls significantly differ (*P* < 0.001; LSD)

f MDD and controls significantly differ (*P* < 0.001; LSD).

Similar levels of depression severity, anhedonia and rumination emerged between CSA + MDD and MDD groups (*all P-*values >0.12) with each clinical group significantly differing from controls (*all P*-values <0.001). Greater sexual abuse was reported by the CSA + MDD group compared to MDD and controls (*P-*values < 0.001). CSA + MDD and MDD showed comparable levels of HRB in past month (*P *= 0.98), which was significantly greater than controls (*all P*-values <0.02). Self-reported state affect measures (PANAS-P and PANAS-N completed pre- and post-scan) were highly correlated for positive (*r*(46) = 0.81, *P* < 0.001) and negative affect (*r*(46) = 0.39, *P* = 0.007), suggesting consistent ranking of participants (Table S6).

### Fronto-striatal group differences

During reward anticipation, no group differences emerged for activation in striatal (Pillai’s trace = 0.28, *F*(16, 74) = 0.75, *P* = 0.73, η_p_^2^ = 0.14) or prefrontal ROIs (Pillai’s trace = 0.32, *F*(8, 82) = 1.94, *P* = 0.06, η_p_^2^ = 0.16).

During reward feedback, no group differences emerged for activation in striatal ROIs (Pillai’s trace = 0.48, *F*(16, 74) = 1.45, *P* = 0.14, η_p_^2^ = 0.24). Significant group differences emerged for prefrontal ROIs (Pillai’s trace = 0.40, *F*(8, 82) = 2.53, *P* = 0.02) with the multivariate effect size estimated at 0.20. *Post hoc* analyses revealed a main effect of group on OFC activation during reward feedback (*F*(2, 45) = 4.67, *P *= 0.02; η_p_^2^ = 0.18). MDD showed lower OFC activation compared to controls [*P *= 0.004, 95% confidence interval (95% CI) 0.23, 1.14], with no differences between CSA + MDD and MDD (*P* =0.13, 95% CI −0.11, 0.80) or controls (*P *= 0.09, 95% CI −0.84, 0.17).

### Moderation: reward anticipation

In Table 2, Δ*R*^2^ represents the change in *R*^2^ associated with introducing the group interaction term (*Clinical Groups vs Controls × ROI beta weights* and *CSA + MDD vs MDD × ROI beta weights*; see also Table S7). Significant Δ*R*^2^ indicates moderation.

**Table 2.** Testing the moderation effects of activation for reward anticipation on the relationship between group and HRB

	Clinical groups *vs* controls × ROI				CSA *vs* MDD × ROI					
	ß	*t*	95% CI		ß	*t*	95% CI		Δ*R*^2^	*F* (2,40)
Right NAcc	5.73	1.14	−4.39	15.85	5.44	1.04	−5.13	16.00	0.04	1.13
Left NAcc	7.81	2.20*	0.65	14.97	12.00	2.67*	2.82	21.07	0.17	5.36**
Right caudate	2.59	0.51	−7.65	12.83	8.94	1.40	−3.95	21.83	0.04	1.03
Left caudate	7.05	1.50	−2.76	6.71	13.96	2.32*	1.78	26.14	0.13	3.79*
Right pallidum	19.49	2.16*	1.22	37.77	12.70	1.54	−4.01	29.42	0.05	3.22
Left pallidum	9.33	1.33	−4.85	23.51	1.53	0.16	−17.78	20.85	0.05	1.22
Right putamen	8.63	1.10	−7.21	24.46	7.76	0.96	−8.61	24.13	0.04	1.15
Left putamen	21.21	3.19**	7.76	34.65	−8.29	−1.17	−22.62	6.05	0.16	5.35**
OFC	−0.78	−0.11	−14.69	13.13	17.80	2.46*	3.16	32.44	0.10	3.03^
mPFC	−11.06	−1.31	−28.07	5.95	−6.58	−0.85	−22.33	9.16	0.04	1.10
Right vlPFC	−6.93	−1.19	−18.70	4.84	−0.66	−0.09	−15.05	13.73	0.03	0.77
Left vlPFC	1.22	0.23	−9.63	12.07	−3.18	−0.66	−12.89	6.54	0.01	0.25

Notes. Note. *F* and Δ*R^2^* reflect the addition of both interaction terms (*Clinical groups vs controls × ROI* and *CSA vs MDD × ROI*) in the moderation (regression) model, i.e. test of highest order unconditional interactions. HRB = high-risk behaviours; ROI = region of interest; CSA = childhood sexual abuse; MDD = major depressive disorder.

* *P* < 0.05.

** *P* < 0.01.

^ *P* = 0.0500.

**Fig. 1.**
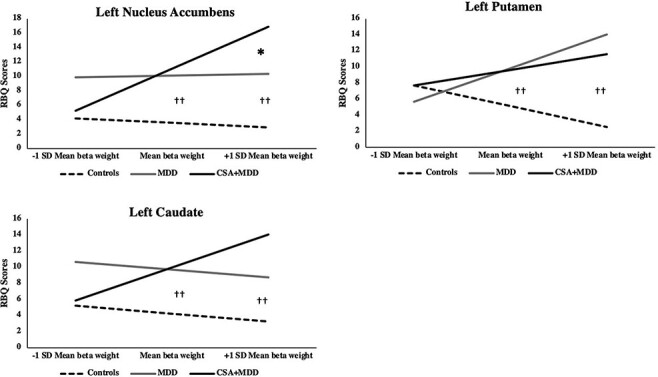
Interaction graphs for reward anticipation. Higher striatal activation in clinical groups predicted greater HRB compared to controls. Higher left NAcc activation predicted greater HRB in depressed adolescents with CSA compared to non-abused peers matched for depression.

For reward anticipation, models with activation in the left NAcc, left caudate and left putamen explained a significant change in variance highlighting their importance as moderators between the groups and HRB (Table 2). Simple slopes analysis with omnibus tests probed interactions by evaluating the conditional effects of group on HRB at low (−1 s.d.), mean and high (+1 s.d.) levels of activation (Figure 1).

Significant interactions emerged for left NAcc activation between Clinical groups *vs* controls and between CSA + MDD *vs* MDD explaining 17% of variance in HRB. While a positive relationship emerged between left NAcc activation and HRB in CSA + MDD, we found no relationship for MDD and a negative relationship for controls. HRB was greater in clinical groups than controls at mean (*B *= 7.04, SE = 1.88, *t *= 3.74, *P* = 0.0006, 95% CI 3.23, 10.84) and high (*B *= 10.67, SE = 2.63, *t *= 4.05, *P *= 0.0002, 95% CI 5.35, 15.98) levels of left NAcc activation. Critically, at high levels of left NAcc activation, CSA + MDD demonstrated more HRB than MDD (*B *= 6.55, SE = 3.06, *t *= −2.14, *P *= 0.04, 95% CI 0.38, 23.72).

A significant interaction emerged for left caudate activation between CSA + MDD *vs* MDD explaining 13% of variance in HRB. A positive relationship was found between left caudate activation and HRB in CSA + MDD, while a negative relationship emerged for MDD and controls. Compared to controls, clinical groups more frequently engaged in HRB at mean (*B *= 5.62, SE = 1.92, *t *= 2.93, *P *= 0.006, 95% CI 1.74, 9.49) and high (*B *= 8.16, SE = 3.14, *t *= 2.93, *P *= 0.006, 95% CI 1.74, 9.49) levels of left caudate activation.

A significant interaction emerged for left putamen activation between Clinical groups *vs* controls but not between CSA + MDD *vs* MDD. A positive relationship emerged between left putamen activation and HRB for CSA + MDD and MDD compared to a negative relationship for controls. Clinical groups more frequently engaged in HRB compared to controls at mean (*B *= 4.60, SE = 1.79, *t *= 2.57, *P *= 0.01, 95% CI 0.99, 8.32) and high (*B *= 10.23, SE = 2.41, *t *= 4.25, *P *= 0.0001, 95% CI 5.37, 15.10) levels of left putamen activation.

### Moderation: reward feedback

For reward feedback, only the model with left putamen activation explained a significant change in variance thus emerging as a moderator between groups and HRB (Table 3).

**Table 3.** Testing the moderation effects of activation for reward feedback on the relationship between group and HRB

	Clinical groups *vs* controls × ROI				CSA + MDD *vs* MDDs × ROI					
	ß	*t*	95% CI		ß	*t*	95% CI		Δ*R^2^*	*F* (2,40)
Right NAcc	1.13	0.65	−2.40	4.67	2.86	1.42	−1.21	6.92	0.05	1.21
Left NAcc	−0.14	−0.07	−4.49	4.20	3.48	1.27	−2.04	9.01	0.03	0.89
Right caudate	−0.09	−0.03	−6.57	6.39	0.85	0.28	−5.26	6.97	0.002	0.04
Left caudate	−0.02	−0.01	−6.53	6.49	−1.21	−0.35	−8.19	5.77	0.003	0.06
Right pallidum	−0.07	−0.02	−7.62	7.49	0.88	0.21	−7.72	9.47	0.0009	0.02
Left pallidum	2.61	1.22	−1.72	6.93	6.47	2.35	0.89	12.04	0.10	3.19
Right putamen	4.46	1.41	−1.93	10.82	1.94	0.49	−5.97	9.85	0.03	1.05
Left putamen	5.08	2.29*	0.60	9.55	5.77	2.02^	−0.0004	11.55	0.14	4.34*
OFC	−2.94	−0.83	−10.09	4.22	2.85	0.78	−4.55	10.25	0.03	0.89
Medial PFC	−5.55	−1.73	−12.03	0.93	−4.37	−1.53	−10.14	1.41	0.09	2.86
Right vlPFC	3.28	1.21	−2.22	8.79	−1.38	−0.54	−6.48	3.73	0.03	0.78
Left vlPFC	2.05	1.34	−1.05	5.14	−2.37	−1.05	−6.94	2.19	0.04	1.09

Notes. *F* and Δ*R*^2^ reflect the addition of both interaction terms (*Clinical groups vs controls × ROI* and *CSA vs MDD × ROI*) in the moderation (regression) model, i.e. test of highest order unconditional interactions. HRB = high-risk behaviours; ROI = region of interest; CSA = childhood sexual abuse; MDD = major depressive disorder.

* *P* < 0.05.

^ *P* = 0.0500.

**Fig. 2.**
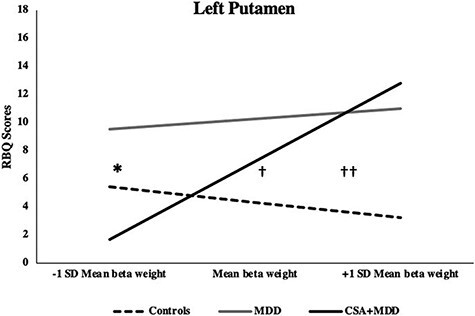
Interaction graphs for reward feedback. Higher activation in left putamen during reward feedback predicted greater HRB in clinical groups compared to controls. Lower left putamen activation in depressed adolescents with CSA predicted absence of HRB compared to non-abused peers matched for depression.

A significant interaction emerged for left putamen activation between Clinical groups *vs* controls and marginally between CSA + MDD *vs* MDD (*P *= 0.0500; Figure 2). Clinical groups showed greater HRB than controls at mean (*B *= 4.44, SE = 1.87, *t *= 2.38, *P *= 0.02, 95% CI 0.66, 8.22) and high (*B *= 8.66, SE = 2.58, *t *= 3.36, *P *= 0.002, 95% CI 3.45, 13.88) levels of left putamen activation. Critically, CSA + MDD demonstrated fewer HRB than MDD (*B *= −7.84, SE = 3.21, *t *= −2.44, *P* = 0.02, 95% CI −14.34, −1.34) at low left putamen activation.

## Discussion

Contrary to our first hypothesis, we did not find differences in fronto-striatal activation to reward in CSA + MDD compared to MDD. However, blunted OFC activation during reward feedback was found in MDD compared to controls. Supporting our second hypothesis, only for CSA + MDD but not MDD, heightened left NAcc activation during reward anticipation was associated with greater frequency of HRB, while decreased left putamen activation during reward feedback was associated with the absence of HRB. Findings highlight the critical role of striatal activation to reward in explaining HRB in adolescents with CSA irrespective of depression. Identifying such ecophenotypic variations associated with CSA is critical to recognize at-risk youth and tailor treatment effectively.

We did not find significant differences in fronto-striatal activation during reward anticipation and feedback in CSA + MDD compared to MDD, suggesting that depressed adolescents may process reward similarly regardless of their experience of CSA. One interpretation could be that the reward system may not be vulnerable to the neurobiological impact of CSA. However, this is not supported by research highlighting blunted reward responses following childhood adversity across the life span (McCrory and Viding, 2015; Teicher and Samson, 2016). Another interpretation is that MDD and CSA are similarly represented by blunted striatal response to rewards, thus cancelling our between-group differences. This would be in line with research indicating blunted striatal reward responses separately in adolescents with MDD (Forbes *et al.*, 2009) and childhood adversity (Hanson *et al.*, 2016) and as an underlying risk factor predicting depression after adversity (Hanson *et al.*, 2015; Dennison *et al.*, 2016). An alternative interpretation could be that key differences in reward processing between MDD and CSA + MDD may only emerge in relation to *specific* situations or behaviours (i.e. heightened striatal activation to reward associated with greater HRB; Poon *et al.*, 2019), rather than representing *generic* differences. Although this explanation is supported by findings from our moderator analyses, further research is needed to test this speculation. Moreover, we found differences between MDD and controls during reward feedback but not reward anticipation. Compared to controls, MDD showed blunted OFC activation, suggesting that they may experience diminished reward value when receiving monetary incentives which may affect decision-making (Rolls *et al.*, 2020). Blunted OFC activation to reward has been found in children and adolescents with depression (Forbes *et al.*, 2006; Xie *et al.*, 2021).

Critically, we found that heightened left NAcc activation during reward anticipation was associated with greater current HRB in CSA + MDD but not in their non-abused peers matched for depression (i.e. symptom severity, episodes and treatment) or controls. While both clinical groups engaged in more HRB than controls, the relationship between striatal activation and HRB for depressed adolescents without CSA was negative or absent. Reward sensitivity may therefore play a critical role in explaining HRB in depressed adolescents with CSA, while HRB in depression only may be driven by other factors (e.g. cognitive control and attentional deviation; Yang *et al.*, 2018). Similarly, only for adolescents with CSA + MDD but not MDD, *low* left putamen activation during reward feedback was linked to an absence of HRB. Blunted striatal response and possibly lower dopamine release to reward may serve as a buffer from engaging in HRB in depressed adolescents with CSA; however, it may also contribute to observed impairments in reward learning following CSA (Pechtel and Pizzagalli, 2013).

This is the first study to show that increased reward sensitivity is specifically linked to HRB in adolescents with CSA, irrespective of depression, lending some support for a CSA ecophenotype. From an evolutionary perspective, it could be argued that when growing up in an adverse environment with few reward cues, heightened reward sensitivity initially forms an adaptive response in which increased mesolimbic activation would allow individuals to detect rewards even in highly stressful contexts (DePasque and Galván, 2017). However, according to the latent theory of vulnerability (McCrory and Viding, 2015) when transitioned to safe contexts, this learned response may be maintained and increases vulnerability to HRB—making them distinct to adolescents with depression only. Understanding ecophenotypic adaptations can provide an important window of opportunity for preventative interventions to be implemented early in development (McCrory *et al.*, 2017).

Our findings are particularly interesting considering the role of reward in risk and resilience. Increased ventral striatal responses to reward have been shown to moderate the relationship between stressful life events and positive affect (Nikolova *et al.*, 2012; Corral-Frías *et al.*, 2016), while reduced ventral striatum activation was associated with anhedonia (Corral-Frías *et al.*, 2015). Dennison and colleagues (2016) found that greater striatal reactivity to reward was associated with resilience to depressive symptoms in adolescents with childhood maltreatment. Heightened reward reactivity may therefore be considered ‘preferable’ following adversity to increase positive affect and avoid depression. However, considering our findings, reward responses may not represent ‘resilience’ per se, but the absence of psychopathology may be accompanied, masked or potentially caused by engagement in HRB.

### Limitations

Given our small sample, both type 1 and type 2 errors are possible, thus findings require replication. Although we used emotional and physical abuse as covariates in the analysis, larger samples will need to confirm the identification of a specific CSA ecophenotype. While no group differences emerged for substance misuse and cigarette smoking in the past month (all *P*-values >0.20), we did not control for smoking or substance use immediately before the scan (>45 min) which may have affected our PFC findings. Due to the habituation of reward responses (Delgado *et al.*, 2000), we selected an early time window of reward-related activation which may have affected the detection of striatal differences in depression. Strengths included focussing on a single abuse type, investigating ecophenotypic variations and applying neural correlates to clinically meaningful behaviours that pose a real-life challenge to clinicians.

### Clinical implications

With the prevalence of CSA and HRB rising, recognizing aberrant reward processes that predict HRB in adolescents is pivotal to identify at-risk youth and increase the effectiveness of interventions. Based on our results, increasing social connectedness with peers may form a promising treatment target following CSA as it has been shown to compensate for aberrant reward processes (Tabibnia, 2020) and to buffer against future stressors (i.e. resilience; Van Harmelen *et al.*, 2017; Fritz *et al.*, 2018). Indeed, Telzer and colleagues (2013) showed that heightened ventral striatal activation to prosocial rewards was associated with longitudinal declines in HRB. Findings highlight that the same ecophenotypic mechanisms conferring vulnerability to HRB (i.e. heightened reward sensitivity) can also serve a protective function against HRB in the right context (i.e. heightened sensitivity to prosocial rewards).

## Conclusion

Adolescence provides an important window of opportunity to shape behaviour to prevent HRB. While we did not find CSA-specific differences in fronto-striatal activation to reward, we found that striatal differences distinguished depressed adolescents with and without CSA in their current use of HRB. Identifying reward-based variations of a CSA ecophenotype provides opportunities for the same neural mechanism conferring vulnerability to HRB to be capitalized on to serve a protective function against HRB.

## Supplementary Material

nsac030_Supp
