# Supplementary material for: Emerging ecophenotype: reward anticipation is linked to high-risk behaviours after sexual abuse
Source: Soc Cogn Affect Neurosci. 2022 Apr 19;17(11):1035–43. doi: 10.1093/scan/nsac030 (PMC9629466; doi:10.1093/scan/nsac030)
Supplement: nsac030_Supp [file nsac030_supp.zip › supp.docx]

**Supporting Information**

**TableS1**
*Region-of-Interest (ROIs) Explored in Current Study*

| **Region-of-Interest** | **Coordinates** | | **Literature**  **Examples** | **Research Area** |
| --- | --- | --- | --- | --- |
| Nucleus Accumbens | | Harvard-Oxford Subcortical Atlas, binarized and thresholded | Braams et al., 2015; Dillon et al., 2009 | High-risk behaviour, childhood adversity |
| Caudate | | Harvard-Oxford Subcortical Atlas, binarized and thresholded | Keren et al., 2018 | Depression |
| Pallidum | | Harvard-Oxford Subcortical Atlas, binarized and thresholded | Dillon et al., 2009 | Childhood adversity |
| Putamen | | Harvard-Oxford Subcortical Atlas, binarized and thresholded | Poon et al., 2019, Dillon et al., 2009 | HRB, childhood adversity |
| Orbitofrontal Cortex | | Harvard-Oxford Cortical Atlas, binarized and thresholded | Xi et al., 202; | Depression |
| Medial Prefrontal Cortex | | Sphere with 25mm radius,  centered on Talairach coordinates: x=0, y=44, z=18 | Forbes et al., 2010 | Depression |
| Ventrolateral Prefrontal Cortex | | Spheres with 10mm radius around MNI coordinates.  Left: x = 46, y = 14, z = 2 Right: x = -54, y = 10, z = 0 | Ambrosia et al., 2018 | High-risk behaviour |

**FigS1**

*Masks Used for (A) Striatal and (B) Prefrontal Cortex Region-of-Interest*

**
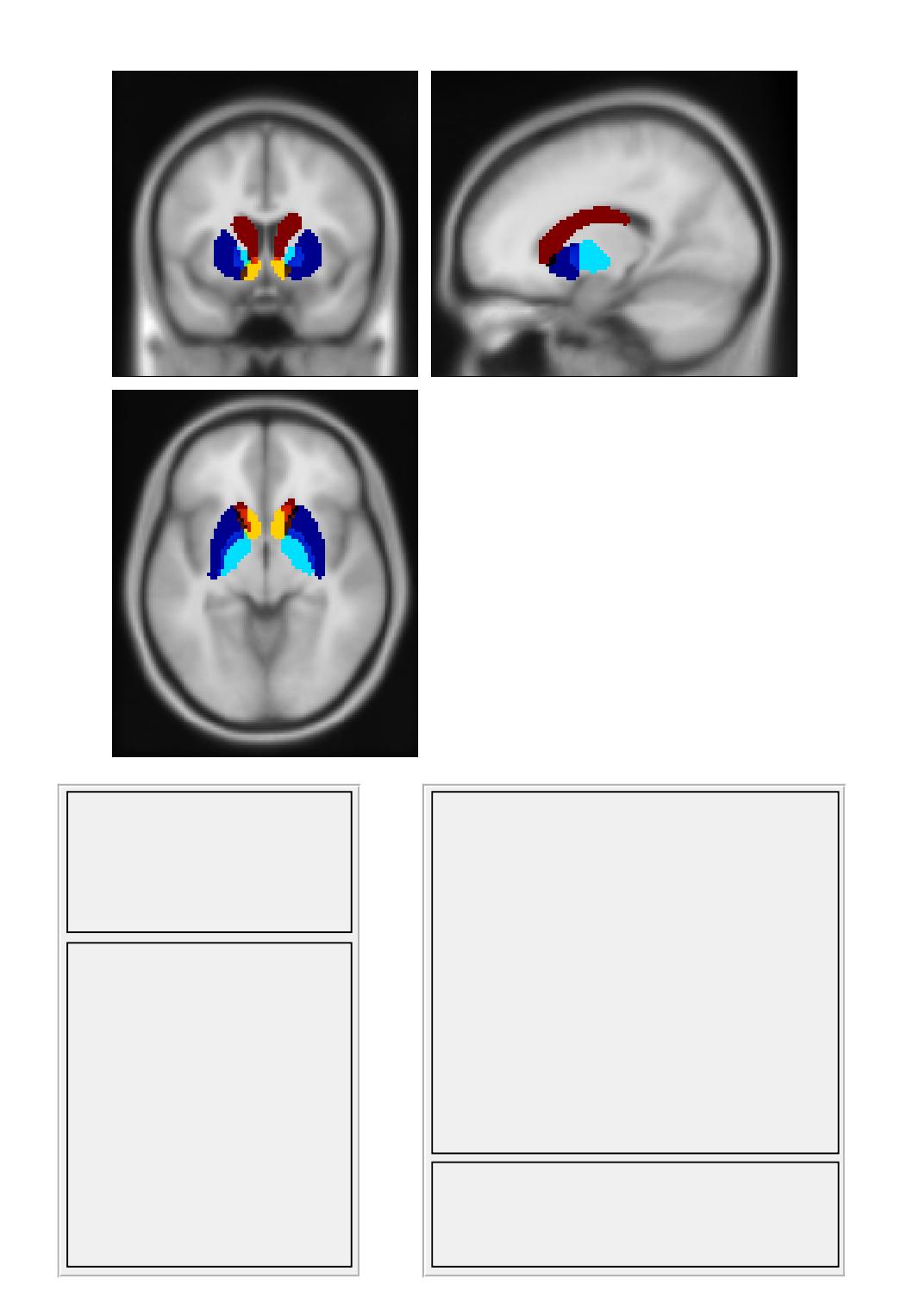

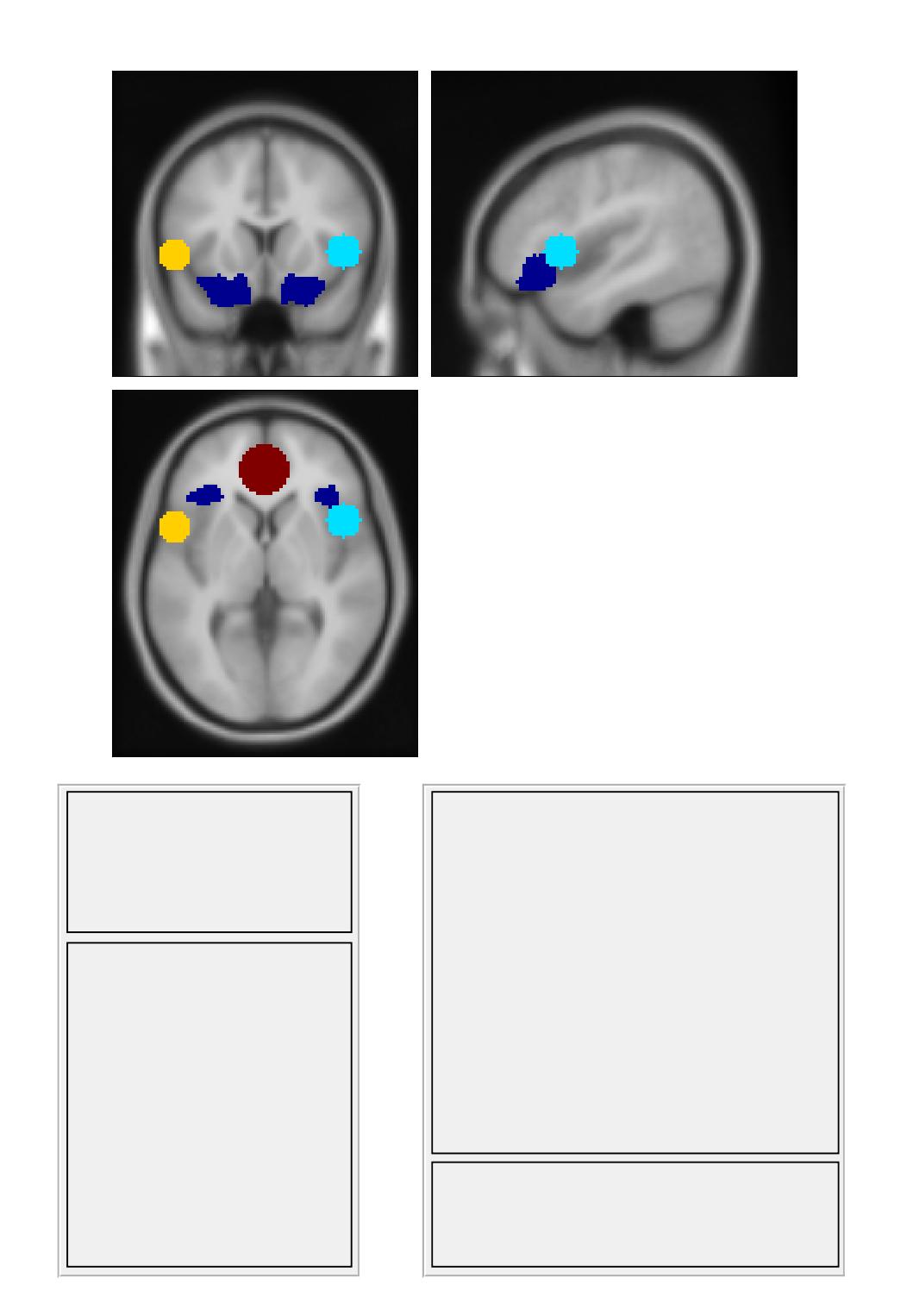
**

Red = Medial prefrontal cortex

Dark blue = Orbitofrontal cortex

Light blue/Yellow = ventrolateral prefrontal cortex

Red = Caudate

Dark blue = Putamen Light Blue = Pallidum

Yellow = Nucleus Accumbens

**A**

**B**

**Methods**

**Participants**

Due to sex-specific differences in brain development, only females were invited to participate (Pechtel & Pizzagalli, 2011). An initial study screen excluded participants who self-reported neurological conditions, mania/hypomania, substance dependence, attention-deficit hyperactivity disorder, psychosis, or were taking medications primarily targeting the dopaminergic system. The study was approved by the UK Health Research Authority. Participants aged 16 or older provided written, informed consent. Parent or guardian provided written consent for participants younger than 16 years.

**Measures**

Participants completed the Child Trauma Questionnaire (CTQ; Bernstein et al., 1994) to assess sexual, emotional and physical abuse that occurred during childhood and adolescence. The sexual abuse CTQ scale consists of 5 items with scores ranging from 5-25 with greater scores indicating greater severity. While scores ≥6 indicate an occurence of CSA, scores ≥ 12 suggest moderate severity and scores of 13 or higher indicate severe CSA. Average sexual abuse subscale scores in our CSA+MDD group (*M*=14.29, *SD*=7.42) indicate moderate to severe severity of CSA. As per inclusion criteria, no sexual abuse (*M*=5.0, *SD*=0.0) was reported by the MDD and control groups.

Participants guessed if an unknown card with a possible value of one to nine was higher or lower than five by pressing the respective buttons on a response box. After guessing, participants saw a “trial type” image indicating a reward (upward arrow) or loss (downward arrow) trial. Participants then saw the actual numerical value of the card followed by a win, loss, or no-change feedback image.

**FigS2**

*Example of a Reward Trial Sequence of the Card Guessing Task (adapted from Forbes et al., 2009).*


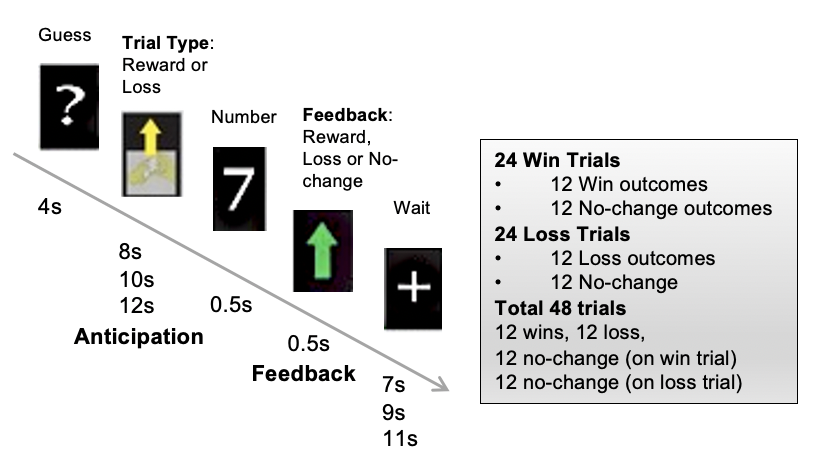


Participants were informed that their performance would determine a real monetary reward that they could keep. Trials were presented in pseudorandomized order with predetermined outcomes resulting in all participants being rewarded a total of £9. Reward anticipation was operationalized as the neural activity when a reward trial was announced. Reward feedback was operationalized as neural activity when participants ‘won’ (green arrow). Trials were separated by a fixation cross which served as implicit baseline for the analysis. Because a priori hypotheses concerned the role of reward processing, analyses did not include loss trials.

**TableS2**

*Reaction Times and Missed Trials on Card-Guessing Paradigm*

|  | **CSA+MDD**  **(*n*=14)** | **MDD**  **(*n*=17)** | **Controls**  **(*n*=17)** | ***F*** | | ***P*** |  |
| --- | --- | --- | --- | --- | --- | --- | --- |
|  |  |  |  |  |  | | |
| RT Total Trials,  *M (SD)* | 1135.27 (356.27) | 1117.46 (287.43) | 990.15 (226.61) | 1.81 | .32 | | |
| RT Reward Trials,  *M (SD)* | 1160.66 (332.35) | 1131.01 (238.78) | 1043.71 (238.78) | 0.67 | .52 | | |
| RT Loss Trials,  *M (SD)* | 1100.90 (415.06) | 1103.94 (64.7) | 936.21 (233.61) | 1.59 | .22 | | |
| Number Missed Trials, *M (SD*) | 3.00 (4.31) | 1.18 (1.01) | 2.71 (4.57) | 1.18 | .32 | | |

**Data Acquisition and Analyses**

Neuroimaging data were collected on a Philips 1.5T whole-body imager (Gyroscan Intera with Explorer gradients) fitted with a 12-channel quadrature brain array coil. The task was presented using an Epson EMP-74 digital projector system and viewed by participants using an angled mirror attached to their head coil. During the task, 38 brain slices were acquired using an interleaved and tilted slice acquisition. T2* weighted echoplanar images were acquired using the following parameters: TR/TE = 3000/45ms; 388 volumes; FOV= 240 mm; Matrix=80x80; voxel size = 3x3x3mm; flip angle = 90°. Functional imaging data for each participant were aligned with their own high-resolution T1-weighted anatomical image for registration into standard space and functional localisation (3D Gradient Echo, TR/TE = 25/4.2ms; 100 volumes, 163 slices, FOV = 230mm; Matrix = 256x204; voxel size = 0.9x.09x1.6mm; flip angle = 30°).

ROI masks (bilateral nucleus accumbens (NAcc), pallidum, caudate, putamen, and OFC) derived from the Harvard-Oxford Subcortical Structural Atlas and were binarized and thresholded to include voxels > 10% probability of being part of the ROI using FSLmaths (Jenkinson, Beckmann, Behrens, Woolrich, & Smith, 2012). Coordinates for the mPFC and vlPFC masks derived from adolescent literature (Ambrosia et al., 2018; Forbes et al., 2010). Neuroimaging data analyses were conducted using Statistical Parametric Mapping (SPM) software, version 12 (London, UK) (<http://www.fil.ion.ucl.ac.uk/spm>) according to **s**standardized pipelines (realignment, slice timing correction, co-registration to T1-weighted image, normalisation, spatial smoothing using a Gaussian kernel of 6 mm full width at half maximum). The Artifact Detection Toolbox was used to identify and de-weight individual whole-brain BOLD fMRI volumes with significant variation in mean volume signal intensity (Version 2015-10; <https://www.nitrc.org/projects/artifact_detect>). Realignment generated six movement regressors for volumes with high motion or artefact, which were included in the general linear model as nuisance covariates, to control for residual movement.

***Aim 1:*** ***Fronto-Striatal Group Differences.***  General linear models were constructed for each participant with an implicit baseline (fixation cross). To increase replicability, we largely modelled the task analysis on previous publications (Caseras, Lawrence, Murphy, Wise, & Phillips, 2013). The anticipatory reward period was divided into an ‘initial’ two-second period and a remaining ‘rest’ period of the anticipation time (varying from 6-10 sec). Only the initial period (2 sec) was analysed as neural responses habituate to reward cues over time, potentially skewing signals when averaged over the entire anticipatory reward period (Caseras et al., 2013; Delgado et al., 2000; Moses-Kolko et al., 2011). Due to the shorter time of ‘feedback’ display, a one-second interval during the delivery of reward was included in the analysis. The canonical haemodynamic response function was used for convolving variables. T-statistics and associated statistical maps were used to depict main effects of task at each voxel for the two contrasts of interest: (1) *reward anticipation*: anticipation of reward vs. baseline and (2) *reward feedback*: win trials of reward delivery vs. baseline.

Separate Multivariate Analysis of Covariance (MANCOVA) for striatal and prefrontal regions were run to determine group-level main effects on the t‐contrast images (anticipation and feedback) with physical and emotional RBQ subscale scores as covariate. Moderate correlations were reported among dependent variables (*TableS3 and TableS4*).

**TableS3**

*Correlations of Striatal and Prefrontal Regions-of-Interest During Reward Anticipation*

|  | 1 | 2 | 3 | 4 | 5 | 6 | 7 | 8 | 9 | 10 | 11 | 12 |
| --- | --- | --- | --- | --- | --- | --- | --- | --- | --- | --- | --- | --- |
| 1. Right NAcc | 1 |  |  |  |  |  |  |  |  |  |  |  |
| 1. Left NAcc | .538** | 1 |  |  |  |  |  |  |  |  |  |  |
| 1. Right Caudate | .636** | .492** | 1 |  |  |  |  |  |  |  |  |  |
| 1. Left Caudate | .567** | .619** | .845** | 1 |  |  |  |  |  |  |  |  |
| 1. Right Pallidum | 0.219 | 0.18 | 0.116 | 0.139 | 1 |  |  |  |  |  |  |  |
| 1. Left Pallidum | 0.196 | 0.052 | .344* | .298* | 0.187 | 1 |  |  |  |  |  |  |
| 1. Right Putamen | .460** | .438** | .375** | .437** | .538** | 0.126 | 1 |  |  |  |  |  |
| 1. Left Putamen | .293* | 0.201 | .340* | .362* | .336* | .659** | .563** | 1 |  |  |  |  |
| 1. OFC | .573** | .566** | .509** | .511** | 0.215 | 0.146 | .554** | .311* | 1 |  |  |  |
| 1. mPFC | .295* | 0.197 | .398** | .335* | -0.165 | -0.076 | 0.235 | -0.052 | .309* | 1 |  |  |
| 1. Left vLPFC | .298* | 0.241 | .327* | 0.252 | 0.027 | 0.021 | 0.16 | 0.145 | 0.155 | .432** | 1 |  |
| 1. Right vlPFC | 0.227 | .303* | 0.146 | 0.152 | -0.014 | -0.063 | 0.069 | -0.086 | 0.256 | .456** | .501** | 1 |

Note. NAcc – nucleus accumbens, OFC – orbitofrontal cortex, mPFC – medial prefrontal cortex, vlPFC – ventrolateral prefrtonal cortex

**TableS4**

*Correlations of Striatal and Prefrontal Regions-of-Interest During Reward Feedback*

|  | 1 | 2 | 3 | 4 | 5 | 6 | 7 | 8 | 9 | 10 | 11 | 12 |
| --- | --- | --- | --- | --- | --- | --- | --- | --- | --- | --- | --- | --- |
| 1. Right NAcc | 1 |  |  |  |  |  |  |  |  |  |  |  |
| 1. Left NAcc | .602** | 1 |  |  |  |  |  |  |  |  |  |  |
| 1. Right Caudate | .624** | .473** | 1 |  |  |  |  |  |  |  |  |  |
| 1. Left Caudate | .297* | .328* | .627** | 1 |  |  |  |  |  |  |  |  |
| 1. Right Pallidum | .297* | 0.254 | .322* | .411** | 1 |  |  |  |  |  |  |  |
| 1. Left Pallidum | 0.079 | 0.176 | 0.182 | .286* | .341* | 1 |  |  |  |  |  |  |
| 1. Right Putamen | .287* | .302* | .451** | .510** | .637** | .636** | 1 |  |  |  |  |  |
| 1. Left Putamen | 0.193 | 0.187 | 0.27 | .346* | .396** | .822** | .715** | 1 |  |  |  |  |
| 1. OFC | .461** | .348* | .409** | 0.281 | 0.194 | 0.253 | .316* | .325* | 1 |  |  |  |
| 1. mPFC | 0.2 | .510** | 0.27 | .310* | 0.126 | .309* | 0.208 | 0.127 | .410** | 1 |  |  |
| 1. Left vLPFC | -0.108 | -0.217 | 0.106 | 0.101 | -0.108 | 0.228 | 0.027 | 0.229 | 0.185 | 0.14 | 1 |  |
| 1. Right vlPFC | -0.074 | -0.084 | 0.051 | 0.164 | 0.081 | 0.228 | 0.198 | .351* | 0.081 | -0.125 | .497** | 1 |

Note. NAcc – nucleus accumbens, OFC – orbitofrontal cortex, mPFC – medial prefrontal cortex, vlPFC – ventrolateral prefrtonal cortex

***Aim 2: Moderation***. The MarsBar toolbox was used to extract beta weights (parameter estimates of activation; arbitrary units) from each subject averaged across all voxels within each ROI. To investigate whether the relationship between CSA and HRB was moderated by fronto-striatal regions during reward anticipation and feedback, simple moderator analyses were performed as hierarchical regressions using PROCESS macro for SPSS (Hayes, 2013). The multi-categorical variable of ‘group’ (Helmert coded as *k-1* groups*: Clinical Groups vs. Controls* and *CSA+MDD vs. MDD)* served as predictor for HRB (RBQ scores), the outcome variable (Hayes & Montoya, 2017). Beta weights were mean-centred to minimise issues with multicollinearity and maximize interpretability (Aiken & West, 1991) and entered as moderators in separate models (one per ROI). In the hierarchical regression, variables were entered as follows: Step 1 - Covariates (CTQ physical and emotional subscale); Step 2 - Predictor (group) and moderator (ROI beta weights) and Step 3 - Interaction terms. To test for moderation, a model comparison approach compared the fit of two models on the outcome variable: specifically, comparing the model with the group interaction (*Clinical Groups vs. Controls x ROI beta weights* and *CSA+MDD vs. MDD x ROI beta weights*) to the one without this interaction. A statistically significant increase in *R^2^* when the group interaction is added constitutes affirmative evidence for moderation. Analysis of simple slopes using omnibus interference test was used to further probe interactions and visualize the models (Hayes & Montoya, 2017).

**Results**

Clinical groups reported similar levels of emotional abuse (*p=.32*) which was greater than for controls (*all p’s<.01*). Clinical groups reported a similar degree of physical abuse (*p=.28*) but only CSA+MDD (*p=.01*) but not MDD (*p=.12*) reported greater physical abuse than controls.

**TableS5**

*Summary of Types of Current Anxiety Presentations, Current Treatment and Medication Use*

| **Current Treatment** | **CSA+MDD (n=14)** | **MDD (N=17)** |
| --- | --- | --- |
| No Current Treatment | 4 | 7 |
| Psychological Therapy | 6 | 3 |
| Medication | 2 | 2 |
| Therapy and Medication | 2 | 5 |
| **Types of Anxiety** |  |  |
| Specific Phobia | 1 | 2 |
| Generalized Anxiety | 5 | 4 |
| Panic Disorder | 0 | 2 |
| **Medication Use** |  |  |
| None | 10 | 10 |
| Medication SSRI | 1 | 6 |
| Medication SNRI ^a^ | 2 | 0 |
| Other | 1 | 1 |

Note. SSRI – Selective Serotonin Reuptake Inhibitor; SNRI – Serotonin-Noreepinephrine Reuptake Inhibitor

^a^ only individuals with low doses (< 150mg/day) of venlafaxine were included. While very high doses can block dopamine reuptake, low doses have been shown to be associated with serotonergic actions (Stahl, 2000).

|  | **CSA+MDD**  **(n=14)** | **MDD**  **(n=17)** | **Controls**  **(n=17)** |
| --- | --- | --- | --- |
| **State Affect** |  |  |  |
| PA-pre, M (SD) | 28.80 (4.99) | 23.35 (6.77) | 29.65 (5.97) |
| NA-pre, M (SD) | 14.66 (3.83) | 13.53 (2.32) | 11.88 (1.90) |
| PA-post, M (SD) | 26.37 (5.83) | 24.00 (9.06) | 5.12 (1.24) |
| NA-post, M (SD) | 12.36 (2.65)^b^ | 11.53 (2.00) | 10.35 (1.00) |
|  |  |  |  |

**TableS6**

*Positive and Negative Affect Data From Participants with a History of Child Sexual Abuse and Depression (CSA+MDD), Depression but no Child Abuse (MDD) and Healthy Controls*

Similar to previous studies (Pechtel & Pizzagalli, 2013), paired t-tests showed a decrease in negative affect in all three groups over course of study: Controls (*t*(16)= 2.72, *p*=.02), MDD (*t*(16)= 3.83, *p*=.001) and CSA+MDD (t(13)= 2.42, *p*=.03). No change of positive affect was shown for controls, MDD or CSA+MDD from pre- to post-MRI scan (*all p’s* <. 06; *TableS6*).

**Moderation: Reward Anticipation and Feedback**

In *TableS7*, *F* and *R^2^* reflect the performance of the models with the inclusion of the interaction in the hierarchical regression.

**TableS7**

*Overall Model Predicting High Risk Behaviour, including Effects of Activation in Regions-of-Interest (Beta Weights), Group (CSA+MDD, MDD or Control) and Interactions Between Group and Activations in Regions-of-Interest (with Emotional and Physical Abuse as Covariates). F and R^2^ reflect the performance of the models with the inclusion of the interaction in the hierarchical regression.*

|  | | **Reward Anticipation** | | | **Reward Feedback** | |
| --- | --- | --- | --- | --- | --- | --- |
|  | *F(*7,40) | | *R^2^* | *F*(7,40) | | *R^2^* |
| Right NAcc | 2.23 | | 0.28 | 1.78 | | 0.24 |
| Left NAcc | **3.55**** | | 0.38 | 1.62 | | 0.22 |
| Right Caudate | 1.71 | | 0.23 | 1.77 | | 0.24 |
| Left Caudate | **2.70*** | | 0.32 | 1.36 | | 0.19 |
| Right Pallidum | **2.61*** | | 0.31 | 1.49 | | 0.21 |
| Left Pallidum | 1.75 | | 0.23 | **3.22**** | | 0.36 |
| Right Putamen | **2.51*** | | 0.31 | 1.93 | | 0.25 |
| Left Putamen | **3.76**** | | 0.40 | **3.34**** | | 0.37 |
| OFC | **2.97*** | | 0.34 | 2.20 | | 0.28 |
| Medial PFC | 1.86 | | 0.25 | **3.06**** | | 0.39 |
| Right vlPFC | 1.59 | | 0.22 | **2.29*** | | 0.29 |
| Left vlPFC | 1.45 | | 0.20 | **2.55*** | | 0.31 |

**Analysis with Treatment as a Covariate: Fronto-Striatal Group Differences**

During reward anticipation, no group differences emerged for activation in striatal (Pillai’s Trace = .35, *F*(16, 72) = 0.96, *p* = .51, η_p_^2^=.18) or prefrontal ROIs (Pillai’s Trace = .29, *F*(8, 80) = 1.67, *p* = .12, η_p_^2^=.14). During reward feedback, no group differences emerged for activation in striatal ROIs (Pillai’s Trace = .48, *F*(16, 72) = 1.43, *p* = .14, η_p_^2^=.24). Significant group differences were shown for prefrontal ROIs (Pillai’s Trace = .38, *F*(8, 80) = 2.34, *p* = .03) with the multivariate effect size estimated at .20. Post-hoc analyses showed a main effect of group on right vlPFC activation during reward feedback (*F*(2, 45)=5.93, *p*=.005; η_p_^2^=.22). CSA+MDD and MDD showed lower right vlPFC activation compared to controls (*p*=.02, 95% CI -1.62, -0.77; *p*=.001, 95% CI -1.74, -0.45, respectively), with no differences between CSA+MDD and MDD (*p* =.48, 95% CI -0.37, 0.76).

**Analysis with Treatment as a Covariate: Moderation Models for Reward Anticipation**

Significant interactions emerged for left NAcc activation between Clinical Groups vs. Controls and between CSA+MDD vs. MDD, whilst controlling for current treatment, physical and emotional abuse, explaining 18% of variance in HRB (17% in previous model). Whilst a positive relationship emerged between left NAcc activation and HRB in CSA+MDD, we found no relationship for MDD and a negative relationship for controls. HRB was greater in clinical groups than controls at mean (B=5.52, SE = 2012, t = 2.61, p=.01, 95% CI 1.24, 9.81 and high levels of left NAcc activation (B=9.42, SE = 2.73, t = 3.46, p=.001, 95% CI 3.91, 14.94). Critically, at high levels of left NAcc activation, CSA+MDD demonstrated more HRB than MDD (B=6.74, SE = 3.01, t = 2.24, p=.03, 95% CI 0.65, 12.84).

A significant interaction emerged for left caudate activation between CSA+MDD vs. MDD, whilst controlling for current treatment, physical and emotional abuse, explaining 13% of variance in HRB (same as in previous model without treatment as covariate). A positive relationship was found between left caudate activation and HRB in CSA+MDD while a negative relationship emerged for MDD and controls. Compared to controls, clinical groups more frequently engaged in HRB at mean (B=4.81, SE = 2.87, t = 2.10, p=.04, 95% CI 1.18, 9.43) and high (B=7.31, SE = 2.91, t = 2.51, p=.02, 95% CI 1.42, 13.21) levels of left caudate activation.

A significant interaction emerged for left putamen activation between Clinical Groups vs. Controls but not between CSA+MDD vs MDD, whilst controlling for current treatment, physical and emotional abuse, explaining 16% of variance in HRB. A positive relationship emerged between left putamen activation and HRB for CSA+MDD and MDD compared to a negative relationship for controls. Clinical groups more frequently engaged in HRB compared to controls at mean (B=4.48, SE = 2.13, t = 2.11, p=.04, 95% CI 0.18, 8.80) and high levels (B=10.11, SE = 2.76, t = 3.66, p=.0007, 95% CI 4.52, 15.69) of left putamen activation.

**Analysis with Treatment as a Covariate: Moderation Models for Reward Feedback**

A significant interaction emerged for left putamen activation between Clinical groups vs. Controls and between CSA+MDD vs. MDD (p=.04; this was lower than the original value). Clinical groups showed greater HRB than controls at only at high levels (B=7.52, SE = 2.74, t = 2.74, p=.009, 95% CI 1.98, 13.08) of left putamen activation (N.B. this was significant at mean levels without the treatment covariate). Critically, CSA+MDD demonstrated fewer HRB than MDD (B=-8.21, SE = 3.22, t = -2.56, p=.01, 95% CI -14.72, -1.71) at low left putamen activation.

**Analysis with Age as a Covariate: Moderation Models**

Significant interactions emerged for left NAcc activation between Clinical Groups vs. Controls and between CSA+MDD vs. MDD explaining 17% of variance in HRB. Whilst a positive relationship emerged between left NAcc activation and HRB in CSA+MDD, we found no relationship for MDD and a negative relationship for controls. HRB was greater in clinical groups than controls at mean (*B*=6.76, SE = 1.93, *t* = 3.51, p=.001, 95% CI 2.87, 10.66 and high levels of left NAcc activation (*B*=10.34, SE = 2.68, *t* = 3.86, *p*=.0004, 95% CI 4.93, 15.76). Critically, at high levels of left NAcc activation, CSA+MDD demonstrated more HRB than MDD (*B*=6.98, SE = 3.12, *t* = 2.24, *p*=.03, 95% CI 0.68, 13.29).

A significant interaction emerged for left caudate activation between CSA+MDD vs. MDD explaining 13% of variance in HRB. A positive relationship was found between left caudate activation and HRB in CSA+MDD while a negative relationship emerged for MDD and controls. Compared to controls, clinical groups more frequently engaged in HRB at mean (*B*=5.35, SE = 1.98, *t* = 2.70, *p*=.01, 95% CI 1.35, 9.36) and high (*B*=7.80, SE = 2.68, *t* = 2.91, *p*=.006, 95% CI 2.37, 13.22) levels of left caudate activation.

A significant interaction emerged for left putamen activation between Clinical Groups vs. Controls but not between CSA+MDD vs MDD. A positive relationship emerged between left putamen activation and HRB for CSA+MDD and MDD compared to a negative relationship for controls. Clinical groups more frequently engaged in HRB compared to controls at mean (*B*=4.75, SE = 1.84, *t* = 2.58, *p*=.01, 95% CI 1.03, 8.47) and high levels (*B*=10.57, SE = 2.55, *t* = 4.14, *p*=.0002, 95% CI 5.41, 15.74) of left putamen activation.

A significant interaction emerged for left putamen activation between Clinical groups vs. Controls and marginally between CSA+MDD vs. MDD (*p*=.0505). Clinical groups showed greater HRB than controls at mean (*B*=4.24, SE = 1.93, *t* = 2.20, *p*=.03, 95% CI 0.34, 8.14) and high levels (*B*=8.50, SE = 2.63, *t* = 3.23, *p*=.003, 95% CI 3.18, 13.81) of left putamen activation. Critically, CSA+MDD demonstrated fewer HRB than MDD (*B*=-7.87, SE = 3.25, *t* = -2.43, p=.02, 95% CI -14.44, -1.31*)* at low left putamen activation.

**Discussion**

Strikingly, all our moderation findings were located in the left striatum. While some studies have found left lateral specificity of functional differences in reward structures associated with childhood adversity (e.g., Dillon et al. 2009), findings overall report mixed laterality effects. One key difference in our study is the specificity of left lateral findings emerged in relation to HRB. This is in line with hemispheric differences in the behavioural expression of emotion which may be driving HRB. Specifically, the left anterior hemisphere has been thought to specialize in processing approach-related or positive emotions while the right anterior hemisphere specializes in withdrawal-related or negative emotions (Teicher & Khan, 2019; Davidson, Ekman, Saron, Senulis, & Friesen, 1990).

**References**

Aiken, L. S., & West, S. G. (1991). *Multiple Regression: Testing and interpreting interactions*. Newbury Park, CA: SAGE.

Ambrosia, M., Eckstrand, K. L., Morgan, J. K., Allen, N. B., Jones, N. P., Sheeber, L., . . . Forbes, E. E. (2018). Temptations of friends: adolescents’ neural and behavioral responses to best friends predict risky behavior. *Social Cognitive and Affective Neuroscience, 13*(5), 483-491. doi:10.1093/scan/nsy028

Braams, B. R., Van Duijvenvoorde, A. C. K., Peper, J. S., & Crone, E. A. (2015). Longitudinal changes in adolescent risk-taking: A comprehensive study of neural responses to rewards, pubertal development, and risk-taking behavior. *Journal of Neuroscience, 35*(18), 7226-7238. doi:10.1523/jneurosci.4764-14.2015

Caseras, X., Lawrence, N. S., Murphy, K., Wise, R. G., & Phillips, M. L. (2013). Ventral striatum activity in response to reward: differences between bipolar I and II disorders. *American Journal of Psychiatry, 170*(5), 533-541.

Davidson, R.J., Ekman, P., Saron, C., Senulis, J. and Friesen, W.V. (1990) Emotional

expression and brain physiology I: Approach/withdrawal and cerebral asymmetry. *Journal of Personality and Social Psychology, 58*, 330-341.

Delgado, M. R., Nystrom, L. E., Fissell, C., Noll, D. C., & Fiez, J. A. (2000). Tracking the

hemodynamic responses to reward and punishment in the striatum. *Journal of Neurophysiology, 84*(6), 3072-3077.

Dillon, D. G., Holmes, A. J., Birk, J. L., Brooks, N., Lyons-Ruth, K., & Pizzagalli, D. A. (2009). Childhood adversity is associated with left basal ganglia dysfunction during reward anticipation in adulthood. *Biological Psychiatry, 66*(3), 206-213.

Forbes, E. E., Hariri, A. R., Martin, S. L., Silk, J. S., Moyles, D. L., Fisher, P. M., . . . Dahl, R. E. (2009). Altered striatal activation predicting real-world positive affect in adolescent major depressive disorder. *American Journal of Psychiatry, 166*(1), 64-73.

Forbes, E. E., Olino, T. M., Ryan, N. D., Birmaher, B., Axelson, D., Moyles, D. L., & Dahl, R. E. (2010). Reward-related brain function as a predictor of treatment response in adolescents with major depressive disorder. *Cognitive, Affective, & Behavioral Neuroscience, 10*(1), 107-118. doi:10.3758/cabn.10.1.107

Hayes, A. F. (2013). *Introduction to mediation, moderation and conditional process analysis: A regression-based approach*. New York, NY: Guilford Press.

Hayes, A. F., & Montoya, A. K. (2017). A tutorial on testing, visualizing, and probing an interaction involving a multicategorical variable in linear regression analysis. *Communication Methods and Measures, 11*(1), 1-30.

Jenkinson, M., Beckmann, C. F., Behrens, T. E. J., Woolrich, M. W., & Smith, S. M. (2012). FSL. *NeuroImage, 62*(2), 782-790.

Keren, H., O’Callaghan, G., Vidal-Ribas, P., Buzzell, G. A., Brotman, M. A., Leibenluft, E., . . . Stringaris, A. (2018). Reward processing in depression: A conceptual and meta-analytic review across fMRI and EEG studies. *American Journal of Psychiatry, 175*(11), 1111-1120. doi:10.1176/appi.ajp.2018.17101124

Moses-Kolko, E. L., Fraser, D., Wisner, K. L., James, J. A., Saul, A. T., Fiez, J. A., & Phillips, M. L. (2011). Rapid habituation of ventral striatal response to reward Receipt in postpartum depression. *Biological Psychiatry, 70(4),* 395-399.

Pechtel, P., & Pizzagalli, D. A. (2011). Effects of early life stress on cognitive and affective function: an integrated review of human literature. *Psychopharmacology, 214*(1), 55-70.

Pechtel, P., & Pizzagalli, D. A. (2013). Disrupted reinforcement learning and maladaptive behavior in women with a history of childhood sexual abuse. *JAMA Psychiatry, 70*(5), 499.

Poon, J. A., Thompson, J. C., Forbes, E. E., & Chaplin, T. M. (2019). Adolescents' reward-related neural activation: Links to thoughts of nonsuicidal self-injury. *Suicide and* Life-Threatening Behavior, 49(1), 76-89. doi:10.1111/sltb.12418

Stahl, S.M. (2000). Essential psychopharmacology neuroscientific basis and practical applications (2^nd^ ed). Cambridge: University Press.

Teicher, M. H., & Khan, A. (2019). Childhood maltreatment, cortical and amygdala morphometry, functional connectivity, laterality, and psychopathology. *Child Maltreatreatment, 24*(4), 458-465.

Xie, C., Jia, T., Rolls, E. T., Robbins, T. W., Sahakian, B. J., Zhang, J., Liu, Z., … & Zhang, Y. (2021). Reward versus nonreward sensitivity of the medial versus lateral orbitofrontal cortex relates to the severity of depressive symptoms. *Biological Psychiatry: Cognitive Neuroscience and Neuroimaging, 6(3),* 259-269.

**Tables and Figures**

**TableS1**. *Region-of-Interest (ROIs) Explored in Current Study*

**TableS2**. *Reaction Times and Missed Trials on Card-Guessing Paradigm*

**TableS3.** *Correlations of Striatal and Prefrontal Regions-of-Interest During Reward Anticipation*

**TableS4.** *Correlations of Striatal and Prefrontal Regions-of-Interest During Reward Feedback*

**TableS5.** *Summary of Types of Current Anxiety Presentations, Current Treatment and Medication Use*

**TableS6.** *Positive and Negative Affect Data From Participants with a History of Child Sexual Abuse and Depression (CSA+MDD), Depression but no Child Abuse (MDD) and Healthy Controls*

**TableS7.** *Overall Model Predicting High Risk Behaviour, including Effects of Activation in Regions-of-Interest (Beta Weights), Group (CSA+MDD, MDD or Control) and Interactions Between Group and Activations* *in Regions-of-Interest (with Emotional and Physical Abuse as Covariates).*

**FigS1.** *Masks Used for (A) Striatal and (B) Prefrontal Cortex Region-of-Interest*

**FigS2**. *Example of a Reward Trial Sequence of the Card Guessing Task (adapted from Forbes et al., 2009).*
